# A novel augmented deep transfer learning for classification of COVID-19 and other thoracic diseases from X-rays

**DOI:** 10.1007/s00521-021-06044-0

**Published:** 2021-04-29

**Authors:** Fouzia Altaf, Syed M. S. Islam, Naeem Khalid Janjua

**Affiliations:** School of Science, Edith Cowan University, Joondalup, WA Australia

**Keywords:** Deep learning, Transfer learning, Dictionary learning, COVID-19, Computer-aided diagnosis, Thoracic disease classification, Chest radiography

## Abstract

Deep learning has provided numerous breakthroughs in natural imaging tasks. However, its successful application to medical images is severely handicapped with the limited amount of annotated training data. Transfer learning is commonly adopted for the medical imaging tasks. However, a large covariant shift between the source domain of natural images and target domain of medical images results in poor transfer learning. Moreover, scarcity of annotated data for the medical imaging tasks causes further problems for effective transfer learning. To address these problems, we develop an augmented ensemble transfer learning technique that leads to significant performance gain over the conventional transfer learning. Our technique uses an ensemble of deep learning models, where the architecture of each network is modified with extra layers to account for dimensionality change between the images of source and target data domains. Moreover, the model is hierarchically tuned to the target domain with augmented training data. Along with the network ensemble, we also utilize an ensemble of dictionaries that are based on features extracted from the augmented models. The dictionary ensemble provides an additional performance boost to our method. We first establish the effectiveness of our technique with the challenging ChestXray-14 radiography data set. Our experimental results show more than 50% reduction in the error rate with our method as compared to the baseline transfer learning technique. We then apply our technique to a recent COVID-19 data set for binary and multi-class classification tasks. Our technique achieves 99.49% accuracy for the binary classification, and 99.24% for multi-class classification.

## Introduction

Deep learning [30] is becoming increasingly popular in medical image analysis [33]. This technology allows to imitate very complex mathematical functions using computational models that can perform intricate decision making with high accuracy. However, deep learning is a data-driven technology. To induce effective computational models in a supervised learning setup, it requires huge amount of annotated training data. In general, this requirement is very hard to fulfil in Medical Imaging [3, 36]. Hence, to leverage deep learning, techniques in the medical imaging domain resort to *transfer learning*. Transfer learning [8, 50] takes a model learned for a *source* domain and applies it to a *target* domain. The source domain is chosen such that it can provide abundant training data to learn an effective model. The parameters of the model are then *fine-tuned* to the target domain using transfer learning with the help of available limited data.

Although useful, transfer learning suffers significantly at the hands of large covariate shift between the source and target domains, and target domain training data scarcity. This is particularly true for medical imaging. Medical image analysis techniques rely on ‘natural’ images to form the source domain [10, 15, 53]. Specifically, they use the models trained on ImageNet data set [12, 34] that contains over one million annotated images of one thousand daily-life object categories. An overwhelming majority of these categories, e.g. carpet, pot, are completely irrelevant for typical medical imaging tasks. Besides, the ImageNet samples are colour images, usually captured in setups far from laboratory settings. Not to mention, the architectures of the models trained for ImageNet data set are designed to discriminate between one thousand classes—a number much larger than a typical medical imaging classification task. These and other such factors not only make transfer learning for medical imaging with these models difficult, they also severely handicap the performance of the overall framework.

In this work, we develop a technique to address above problems by significantly boosting the transfer learning performance for medical image classification task. Specifically, we aim at the settings where the target domain data is not only scarce but also has a different modality than the source domain. We consider ImageNet models trained on colour images and use those to classify thoracic diseases with chest radiography greyscale images. We choose the ImageNet models (trained on natural images) as the source models following the common practice of transfer learning in medical image analysis. On the other hand, our choice of thoracic disease classification as a test bed is based on the fact that these diseases are considered a major health threat around the globe [60]. Moreover, availability of the Chest X-ray 14 data set [62] allows us to clearly establish the effectiveness of our approach by considerably varying the amount of available data for our target domain. Additionally, we are able to demonstrate the effectiveness of our technique for a practical contemporary problem of COVID-19 detection using radiography.

In the proposed technique, instead of following the norm of simply fine-tuning an ImageNet model with the samples of our target domain and account for the data modality difference with image preprocessing, we propose a sophisticated framework that tunes a collection of source domain models to the target domain through a hierarchical learning process. For the source domain models, we use DenseNet [21], ResNet [17], VGG-16 [65] and Inception-V3 [52] models. The framework meticulously extends the original models to automatically account for the data modality differences during training. Eventually, the models are used in an ensemble to make the predictions. These predictions are further augmented by the output of an ensemble over dictionaries [57] formed by the features of data samples extracted through our models. We combine both sparse and dense representations [2] for the dictionary ensemble to augment the output of deep learning model ensemble. We thoroughly evaluate our technique with the challenging multi-class classification scenario of thoracic diseases. Our experiments with Chest X-ray14 data sets show that the overall technique results in a large gain over the performance of the best fined-tuned model under typical transfer learning setup when both techniques use the exact same limited training data. At the same time, our technique results in $99.49\%$ binary class classification accuracy and $99.24\%$ multi-class classification accuracy for the recent COVID-19 data set.

The remaining article is organized as follows. In Sect. 2, we review the related literature. The proposed technique is discussed in Sect. 3. We present experimental results in Sect. 4. The article concludes in Sect. 5, also discussing future work.

## Related work

Since we choose thoracic disease classification with chest radiography as our test bed, we mainly focus on deep learning based techniques only for this problem in this section. Deep learning is becoming increasingly popular for the said problem [49]. For instance, Wang et al. [62] developed a weakly supervised framework for multi-label classification and localization of thoracic diseases and reported results for eight common pathologies on chest X-ray8 data set. They used different ImageNet models for abnormal finding classification and localization. Later, Li et al. [31] proposed a unified approach for disease identification and localization with limited annotated data. They employed the Multiple Instance Learning (MIL) formulation, which helped them to improve the performance as compared to the baseline models of ResNet and DenseNet. Zhou et al. [66] proposed a weakly supervised adaptive DenseNet-169 for the thoracic disease identification and classification in chest radiographs.

Rajpurkar et al. [45] proposed a 121-layered CNN model named ChexNet which is claimed to achieve human level performance on the F1 metric for pneumonia detection. However, it requires training on the large-scale Chest X-ray14 data set [62]. Wong et al. [64] proposed a deep learning-based framework using an ImageNet model Inception-ResNet-V2 for normal vs abnormal classification of 3217 chest X-ray images. Wang et al. [60] proposed ChestNet model which consists of a classification branch and an attention branch for computer-aided diagnosis of thoracic disease on CXR images [62]. They used ResNet-152 which is pretrained on ImageNet data set. The classification branch assists as a uniform feature extractor, and attention branch exploits the correlation between the class label and pathological abnormalities through analysing the feature maps learned in the classification branch. Ho et al. [19] proposed a technique for multiple feature integration. They used pretrained model DenseNet-121 for localization and integrated the extracted shallow features (Scale-Invariant Feature Transform (SIFT), Local Binary Pattern (LBP), Histogram Oriented Gradients (HOG)) and deep (CNN) features for classification of 14 Thoracic disease in chest X-rays. Similarly, Lakhani et al. [29] used AlexNet and GoogLeNet to classify pulmonary tuberculosis. In [63], the authors proposed the TieNet model for text representations of distinctive image extraction. They used the TieNet for classification of X-ray images using image features and text extracted from analogous reports. Furthermore, they utilized the TieNet for CXR reporting system that simulates reporting and outputs disease classification with a precursive report.

From the transfer learning viewpoint, this framework is often used by medical imaging community to address the issue of small data size. Grickshik et al. [16] is one of the first contributions to use transfer learning with pretrained CNNs for image classification [46] while learning from relatively small data set for object detection [13]. Recently, Raghu et al. [44] studied transfer learning for medical images and found that it is challenging to directly take advantage of ImageNet models with transfer learning for the tasks like thoracic disease classification. Da et al. [39] used features extracted from a set of ImageNet models for lung nodule classification in CT images. They used the extracted features to train a list of multi-class classifiers, including MLP, and studied their performance. Nevertheless, they neither consider model ensembles, nor they contributed towards thoracic disease classification. Behzadi et al. [6] used a pretrained ImageNet models for the detection of consolidation in Pediatric Chest X-ray images. They tuned their problem-based ChestNet by removing a few pooling layers and used image preprocessing to cater for model generalization. With the worldwide outbreak of COVID-19, studies on X-ray images have been conducted. Aplostolopoulos et al. [5] transferred some existing object classification models into the COVID-19 classification area. They compared five currently existing models, namely, VGG-19 [51], MobileNet v2 [47], Inception [52], Xception [7], and Inception ResNet v2 [9]. VGG-19 outperforms the other models and has an accuracy of $98.75\%$ in the two-class classification scheme and $93.48\%$ in the three-class classification scheme. Kumar et al. [27] used ResNet-152 to extract features with seven traditional machine learning classifiers, including logistic regression, nearest neighbours, decision tree, random forest, AdaBoost classifier, naive Bayes, and XGBoost classifier. This model has an accuracy of $97.7\%$ on the XGBost classifier. Farooq et al. [14] developed COVID–ResNet, a deep learning framework that aims to classify COVID-19. This framework is highly sensitive to normal $96.58\%$ and COVID-19 $100\%$ classes.

## Proposed technique

**Fig. 1:**
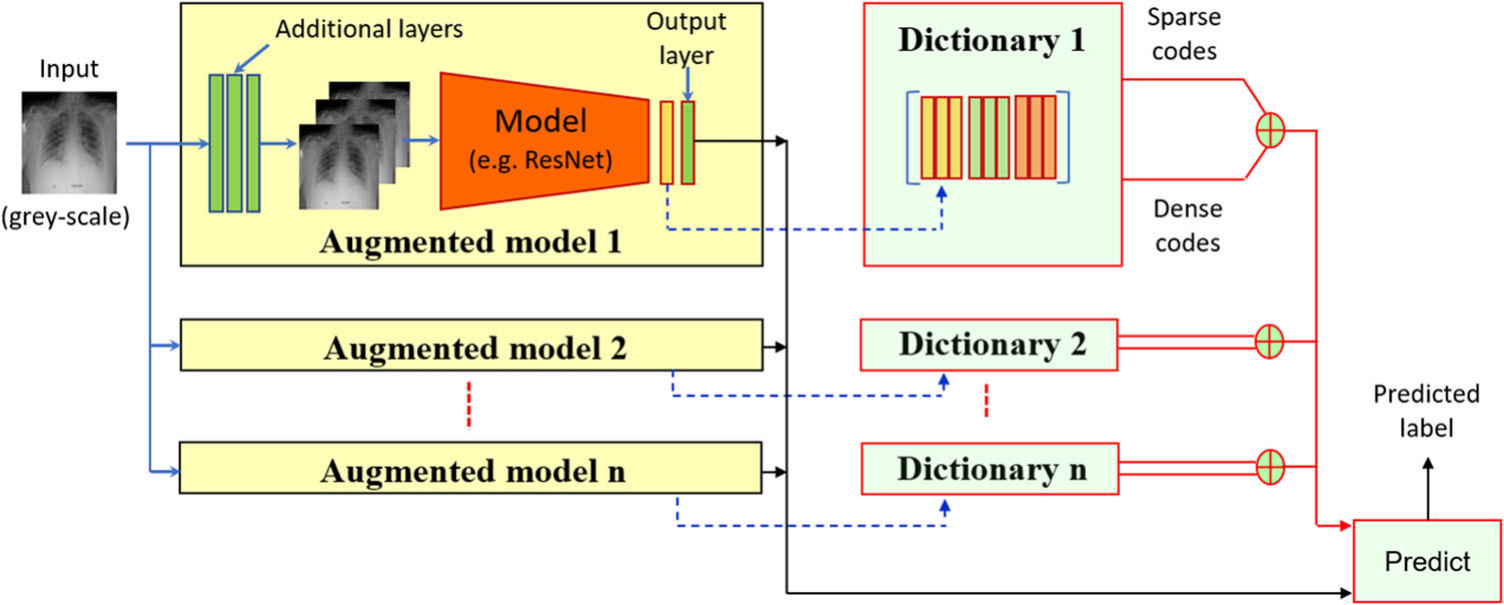
Schematics of the proposed technique: A set of natural (colour) image deep learning models are augmented with additional input and modified output layers. The augmented models are hierarchically fine-tuned with limited (greyscale) images of chest X-rays. Features extracted from augmented models are also used as dictionaries to compute dense and sparse representations of unseen samples. Outputs of model ensemble and dictionary codes are combined to predict output labels

For the transfer learning techniques reviewed in the previous section, and other such techniques in general, the domain transfer is mostly carried out with a rather simplistic fine tuning of the original (i.e. source domain) model using the target domain data. In a sharp contrast, we propose a much more sophisticated transfer learning that not only systematically changes the architecture of the original network, but also hierarchically fine-tunes it on an augmented target domain data. Moreover, we use an ensemble of the modified networks to make a prediction. The prediction mechanism is further augmented by an ensemble of a dictionary-based classification mechanism. Our technique is able to exhibit a significant performance gain over the conventional transfer learning using exactly the same limited training data of the target domain data. A schematic of the overall technique is illustrated in Fig. 1. Below we provide details related to each component of our technique.

### Source domain model selection

Following the common convention of employing natural images as the source domain for medical image transfer learning, we consider the networks trained on ImageNet as the source domain models in this work. To that end, we choose DenseNet201 [20], ResNet50 [17], Inception-V3 [52] and VGG-16 [65] models. As can be seen in Fig. 1, we use these models in an ensemble. Hence, our selection is based on the criterion of increasing the architectural diversity of the underlying networks, thereby increasing the representation power of the ensemble. We refer interested readers to the original works for the exact details of the original network architectures. Below we summarize the major insights that resulted in our final selection of the models.

Among the mentioned models, VGG [51] has a very systematic architecture that gradually compresses the size of the input image/features as we go deep into the network, but keeps increasing the number of convolutional filters to account for this reduction in the feature size. This results in an increasing number of feature maps in the later layers of the network, which endows strong representation power to the overall model. Different from VGG, the strength of ResNet [17] comes from *skip connections* that allow copying residue of feature maps from one layer to a subsequent layer. It is now a common knowledge that these connections are able to significantly improve the network’s representation power. As compared to ResNet, VGG is completely void of skip connections, which makes its internal representation quite different from ResNet.

DenseNet [20] builds on the insights of ResNet and introduces dense skip connections by connecting a layer to all subsequent layers through skip connections. This, along with further architectural changes, significantly alters the internal representation of DenseNet from the ResNet. Hence, we also include this network in our pool of models. One important aspect to note is the use of suffixes with the network names in the preceding paragraphs. Crudely speaking, ‘16’, ‘50’ and ‘201’ refer to the number of layers in the networks. As can be seen, the chosen models go from relatively shallow to very deep in terms of the number of layers. This is intentional, as it results in more architectural diversity.

Different from VGG, ResNet and DenseNet the architecture of Inception-V3 [52] mainly relies on inception blocks for its representation prowess. These blocks are not available in any of the aforementioned networks. Consequently, the architecture and the internal representation of Inception-V3 are considered very different from those networks. This makes Inception-V3 a good candidate for our pool. As can be noticed, our selection of models is methodical. All ImageNet models are trained on the same data set (i.e. ImageNet). This makes the diversity of their internal representation a direct function of their architectures. Hence, by employing models of diverse architectures, we maximize the representation capacity of our ensemble.

### Deep model augmentation

The most common issue for transfer learning is the mismatch between the dimensions of input expected by the network and the actual dimensions of the target domain samples. Moreover, the number of class labels to be predicted at the output may also differ for the target domain. To resolve the latter, we employ the common strategy of replacing the ‘fully connected’ and ‘softmax’ layers of the original networks with N-neuron layers of the same types, where ‘N’ is the number of output classes of the target domain. For the former, we augment the input stage of the networks with additional convolutional layers. These layers serve two purposes in our models. (a) They allow us to use greyscale single-channel images as inputs to the networks that are originally pretrained on three-channel colour images. (b) We are also able to use images twice as large as the input images originally expected by the model.

In above, (a) is important because the images for chest radiography are grey scale. Generally, the difference in the number of channels of greyscale and colour images is handled by the existing methods by either three-fold stacking of the single channel image, or using primitive image processing techniques. In contrast, we let our model ‘learn’ this transformation automatically from the target domain itself using end-to-end training. Moreover, due to (b), we are able to take advantage of additional information in larger input images. The large images also get compressed automatically by the added convolutional layers in our augmented networks. For each of the used model, we summarize details of the added layers in Table 1. According to the table, the ‘Original’ input layer gets replaced by the ‘Modified’ block of layers, which expects a lager ‘Input’ size. It can be noticed that the dimensions of the output ‘Activations’ of the ‘Modified’ layers match the dimensions of the ‘Input’ of the ‘Original’ layer. Hence, an added block seamlessly combines with the original model. The convolutional kernel size in each ‘Modified’ block is kept similar to the kernel size of the first convolutional layer of the original network. The stride and number of filters are adjusted to match the input dimensions expected by the first convolutional layer of the original network. Similarly, the Batch-Normalization operation and ReLU activations are also applied based on their application in the original architectures.

**Table 1:** Network adaption for transfer learning at input stage

Network	Original	Input	Modified	Input	Activations
DenseNet201	Input_1	224$\times$224$\times$3	Input_grey	448$\times$448$\times$1	224$\times$224$\times$3
			Conv 7, 3, [2,2]		
			Batch-N, ReLU		
ResNet50	Input_1	224$\times$224$\times$3	Input_grey	448$\times$448$\times$1	224$\times$224$\times$3
			Conv 7, 3, [2,2]		
			Batch-N, ReLU		
Inception-V3	Input_1	299$\times$299$\times$3	Input_grey	598$\times$598$\times$1	299$\times$299$\times$3
			Conv 3, 3, [2,2]		
			Batch-N, ReLU		
VGG-16	Input	224$\times$224$\times$3	Input_grey	448$\times$448$\times$1	224$\times$224$\times$3
			Conv 3, 3, [2,2]		
			Batch-N, ReLU		

‘Original’ names of the altered layers are given along the ‘Input’ dimensions expected. For the ‘Modified’ network, *conv K, F, [S,S]* indicates a convolutional kernel with kernel size $K \times K$, with *F* number of filters and a stride of *[S, S]*. The activations of convolutional layer are batch-normalized [22], indicated by ‘Batch-N’, followed by ReLU activations [37]. The output ‘Activations’ of the modified layer are given in the last column. At the output stage, the fully connected layers are modified to have *N* neurons instead of 1000, where *N* is the number of output classes considered

### Deep model training

We train our augmented deep models individually using a hierarchical three-step strategy. In the first step, we freeze the inner layers of the network and train only the augmented input layers and the modified output layers for five epochs using learning rate 0.001 with Adam optimizer [26]. This training is done using the original training data of our target domain. The objective of this step is to reach a reasonable initialization point for the subsequent training steps. In the second step, we reduce the learning rate ten times and conduct five more training epochs; however, this time we use augmented training data. We give details on data augmentation in the next paragraph. In the third step of model training, we further reduce the learning rate ten times and run five more epochs using the augmented data. However, this time we let the complete network get fine-tuned to smooth out any abrupt weight variations between the original and augmented layers. The last step is allowed to modify weights of the whole network, albeit slightly. We use the default parameter settings of the Adam optimizer for training, except for the learning rate, which is varied as discussed above.

In our implementation, we employ cropping, rotation and flipping as the data augmentation strategies. For cropping, we select the inner $850\times 850$ block of the original $1024\times 1024$ image and then resize the cropped image back to $1024\times 1024$. For rotation, we use a random angle of rotation between −7 to 7 degrees. This is based on the observation that chest radiographs are often tilted in the same range. We only apply horizontal flip. To augment the training data, an additional copy of the original sample gets modified by each of the aforementioned transformations with 0.3 probability in a sequential manner. This results in almost doubling our training data synthetically.

### Representation augmentation with dictionaries

Within the broader field of Machine Learning, deep learning is a representation learning technique. Another popular technique for representation learning is known as ‘dictionary learning’ [57] that represents data with the help of an over-complete basis. Put simply, given an (over-complete) basis $\mathbf{D}$—a.k.a. dictionary—for an input sample $\mathbf{y}$, it strives to compute a representation vector $\varvec{\alpha }$ such that $\mathbf{y} \approx \mathbf{D} \varvec{\alpha }$. The representation $\varvec{\alpha }$ can be subsequently used for further analysis. It has been shown that this technique can boost the performance of deep learning representation [1]. Hence, we further augment our framework with this technique to achieve maximum performance with minimal training data of the target domain.

Concretely, given an augmented fine-tuned model, we forward pass all original training samples through it and record the activations of the layer before the softmax layer. For each model, we use these activations to construct the dictionary $\mathbf{D}$. Essentially, each column of the dictionary thus constructed is a feature of a training sample, as extracted from our modified model. With ‘*n*’ models used in our ensemble, we create ‘*n*’ such dictionaries with the same training data. Each of these dictionaries is unique because their columns are based on different representations resulting from different models. These dictionaries eventually get used in the classification stage of our framework.

#### Representation computation with dictionaries

Whereas we give complete details of the classification stage of our technique in Sect. 3.5, for clarity, it is imperative to discuss the representation computation of testing samples with dictionaries here.

In order to eventually classify a test sample, we forward pass it through each of our augmented fined-tuned networks. Assume that $\widetilde{\mathbf{y}}$ denotes the feature extracted for the test sample from one of our models, where feature extraction follows the same procedure that we use for dictionary creation. We use the dictionary $\mathbf{D}$ for that model to solve the following two optimization problems to compute two new representations for the test sample:

1 $$\begin{aligned} \varvec{\alpha }^d= &\, \underset{\alpha }{\text {argmin}}\,|| \widetilde{\mathbf{y}} - \mathbf{D}\varvec{\alpha }||_2 + \lambda ||\varvec{\alpha }||_2, \end{aligned}$$

2 $$\begin{aligned} \varvec{\alpha }^s= &\, \underset{\alpha }{\text {argmin}}\,|| \widetilde{\mathbf{y}} - \mathbf{D}\varvec{\alpha }||_2\,\,\text {s.t.}\, ||\varvec{\alpha }||_0 \le k \end{aligned}$$

where ‘$\lambda$’ is a regularization constant, $||.||_p$ denotes the $\ell _p$-norm of a vector and ‘*k*’ is a predefined constant. The first equation seeks to solve $\widetilde{\mathbf{y}} = \mathbf{D} \varvec{\alpha }^d$ in a regularised least squares manner. We can compute a closed form solution for that by letting $\varvec{\alpha }^d = (\mathbf{D}^{\intercal } \mathbf{D} + \lambda \mathbf{I})^{-1} \mathbf{D}^{\intercal } \widetilde{\mathbf{y}}$, where $\mathbf{I}$ is the identity matrix. The resulting $\varvec{\alpha }^d$ is a ‘dense’ vector in the sense that nearly all of its coefficients will have nonzero values. Hence, we use the superscript ‘*d*’ in $\varvec{\alpha }^d$. For $\varvec{\alpha }^s$, the external constraint $||\varvec{\alpha }||_0 \le k$ forces $\varvec{\alpha }^s$ to have at most ‘*k*’ nonzero coefficients, thereby making this vector ‘sparse’—indicated by the superscript ‘*s*’. It has been shown by Akhtar et al. [2], that both sparse and dense representations can work hand-in-hand to make a cumulative representation achieved as $\varvec{\alpha } = \varvec{\alpha }^d + \varvec{\alpha }^s$ more discriminative than any of these representations alone. We capitalize on this observation and use the more discriminative representation in our final classification. We use the Orthogonal Matching Pursuit (OMP) strategy [42] to compute the sparse representation $\varvec{\alpha }^s$. We note that the concept of using deep learning features to construct dictionaries was first introduced in [1]. However, our technique is different from [1] in that we are strictly concerned with transfer learning for which our base deep learning model is also augmented and fine-tuned to the target domain. In [1], transfer learning is not considered. Additionally, the features used by [1] are only employed for initializing a dictionary that later gets adapted to the training data of the same domain for which the deep model is trained. Moreover, [1] also does not consider combining the sparse and dense representations as proposed in this work, which makes our contribution significantly different from [1].

### Classification

In order to classify a test sample, we forward pass it through each of the models in our ensemble and record the activations of their softmax layers. We also record the activations of the layers right before the softmax to compute the cumulative representation $\varvec{\alpha }$, as discussed in Sect. 3.4.1. Recall that we construct the columns of dictionaries involved in computing $\varvec{\alpha }$ with the features of annotated training data. This allows us to specify a class label associated with each column of a dictionary. Algebraically, those labels also get associated with the corresponding coefficients of $\varvec{\alpha }$. We take advantage of this observation and compress our representation $\varvec{\alpha }$ by integrating the components of this vector for each class, resulting in a vector $\varvec{\alpha }^{\text {compressed}} \in \mathbb R^{\ell }$, where ‘$\ell$’ denotes the total number of classes involved in our classification. From theoretical viewpoint, $\varvec{\alpha }^{\text {compressed}}$ encodes a cumulative correlations between the test sample and the training data of each class. We normalize this vector to have unit magnitude, and use the resulting vector $\hat{\varvec{\alpha }}^{\text {compressed}}$ with the softmax layer activations of our deep models as follows:

3 $$\begin{aligned} {\text {Label}} = \text {Max-coeff}\Big (\sum \limits _{i = 1}^n (\text {softmax}_i + \hat{\varvec{\alpha }}^{\text {compressed}}_i)\Big ), \end{aligned}$$

where Max-coeff(.) is a function that finds the index of the largest coefficient of a vector and ‘*n*’ is the total number of used deep learning models—4 in this work. It is worth noticing that Eq. (3) can be interpreted as maximization of the Expected value of probability distributions over the predicted test label where the distributions are estimated under diverse representation learning tools.

## Experiments

We use thoracic disease classification as the test bed for our technique. Below we give details of the experiments that establish the effectiveness of our technique.

**Table 2:** Results summary on Chest X-ray14 data set: conventional transfer learning (TL) with DenseNet201 is the ‘Baseline’

Models	Spec.$\%$	Sens.$\%$	F1$\%$	Acc.$\%$	ERR$\%$	$\dagger$Acc.$\%$	$\dagger$Gain$\%$
Baseline (TL)	89	80	–	83.33	–	16.76	–
DenseNet201 (Den.)	94	55	46	89.65	37.91	48.27	188.0
Den+VGG	94	55	47	90.00	3.38	50.03	3.65
Den+VGG+Res	94	58	51	90.63	6.29	53.17	6.27
Den+VGG+Res+IV3	95	60	53	91.03	4.27	55.17	3.76
Proposed	95	60	53	91.38	3.90	56.90	3.13

‘Dense’ denotes DenseNet201 augmented with our technique. Similarly, ‘VGG’, ‘Res’ and ‘IV3’ are augmented versions of VGG-16, ResNet50 and Inception-V3 using our method. ‘Full ensemble’ is the final technique. The Error Reduction Rate (ERR) is computed using Accuracy (Acc.) of two consecutive rows. The $\dagger$Gain is computed with two consecutive rows of $\dagger$Acc

### ChestX-ray14 data set

For experiments, we use publicly available large-scale Chest X-ray14 data set [62]. In total, the data set contains 112,120 frontal chest X-ray images from 30,805 unique patients with 14 disease labels. The full data set has 51,708 images with single or multiple disease labels and 60,412 images with no disease. The original data has a split list with 86,524 images for training and validation and 25,596 for testing. The original resolution of the sample images provided by the data set is 1024x1024. For each augmented network used in our technique, we resize the images to the input dimensions noted in Table 1 using cubic interpolation after data augmentation, where applied.

In our experiments, we consider the scenario where a sample from the data set can contain at most one disease that needs to be correctly classified. This makes our contribution specific to single label multi-class classification. We note that this work is not concerned with multi-label classification, and that will form part of our future work. The chosen setup resulted in identifying 10 classes in the training data with a reasonable amount of samples for conclusive evidence of the effectiveness of our technique. For each of the ten classes, we sample 775 images allowed by the data set, resulting in 7,750 training samples per experiment. The class labels for the used ten classes include Atelectasis, Cardiomegaly, Effusion, Infiltration, Mass, Nodule, Pneumothorax, Consolidation, Pleural Thickening, and No finding. For testing, we select random 3000 samples from the same labels using the testing data of Chest X-ray14. It is worth mentioning that none of the patients in the training data overlaps with the patients of testing data in Chest X-ray14, which makes the data set specifically challenging for transfer learning.

### COVID-19 data set

The second data set used to establish the effectiveness of our technique comprises two publicly available data sets related to COVID-19. These data sets include the COVID-19 radiography data set [54] that is available at kaggle repository which is developed using images from various open access sources, and COVID-19 image data [11] which is separately available for research purpose.

**Fig. 2:**
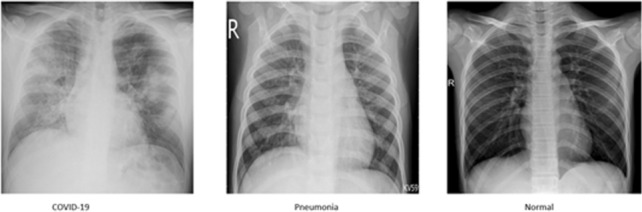
Samples of chest X-ray images from COVID-19 data set

From these data sets, we selected a total of 657 chest X-ray images consisting of 219 images each for COVID-19, viral pneumonia and normal X-rays. Figure 2 shows representative samples of our data set. To be compatible with the models, the data were resized to $448\times 448$ and $598\times 598$. The data set was split into 80% training and 20% testing sets. For the transfer learning, cross-validation revealed an optimal batch size of 16 and only 7 epochs were necessary to fine-tune the model with learning rate 0.001. We fine-tuned the model obtained from our experiments with the Chest X-ray14 data set.

### Evaluation metrics

We employ standard evaluation metrics used by medical imaging and computer vision community to establish the effectiveness of our technique. Recall that our source domain is natural images, which is relevant to computer vision community. Hence, we find it more informative to also include the metric scores used for computer vision tasks.

Let TP, TN, FP and FN denote the number of true positive, true negative, false positive and false negative predictions. We use the following definitions of Specificity (Spec.), Sensitivity (Sens.) and F1-Score:

4 $$\begin{aligned} \text {Spec.}= & \frac{\text {TN}}{\text {TN} + \text {FP}},\,\,\text {Sens.} = \frac{\text {TP}}{\text {TP + FN}}, \end{aligned}$$

5 $$\begin{aligned} \text {F1-Score}= & 2 \times \frac{\text {PPV} \times \text {TPR}}{\text {PPV} + \text {TPR}} \end{aligned}$$

where PPV $=$ TP/(TP+FP) and TPR $=$ TP/(TP+FN). Based on these metrics, we use the definition of Accuracy (Acc.), as commonly adopted by the medical imaging community [28] as:

6 $$\begin{aligned} \text {Acc.} = \frac{\text {TP} +\text {TN}}{\text {TP} +\text {TN} + \text {FP} + \text {FN}} \times 100\%. \end{aligned}$$

We also use $\dagger$Accuracy ($\dagger$Acc.), which is the definition of accuracy commonly used in computer vision community [1] for single-label multi-class classification problems:

7 $$\begin{aligned} \dagger \text {Acc.} = \frac{\text {TP}}{\# \text {of total test samples}} \times 100\%. \end{aligned}$$

It should be noted that the two definitions can amount to different accuracies for the same set of predictions. We often refer to Acc. as the binary classification accuracy and $\dagger$Acc. as the multiclass classification accuracy.

**Table 3:** Computational times for the used models: step 1 is the training of input and output layers with frozen inner layers for 5 epochs with learning rate 0.001

Models	Training time			Testing time (milliseconds)
	Step1	Step2	Step3	
DenseNet201	3 h 20 m 5 s	6 h 15 m 20 s	7 h 29 m 5 s	6
VGG	0 h 55 m 15 s	1 h 51 m 25 s	2 h 5 m 20 s	8
ResNet	0 h 47 m 20 s	1 h 26 m 15 s	1 h 35 m 10 s	6
InceptionV3	1 h 14 m 25 s	2 h 23 m 15 s	2 h 31 m 15 s	6

Step 2 is the training on augmented data with the model resulting from step 1, using learning rate 0.0001 for five epochs. Step 3 is the training on augmented data with learning rate 0.00001 and fine tuning the complete model weights for 5 epochs. We also include the dictionary computation time in this stage. Test time is for a single image, including sparse coding stage

**Table 4:** Results of individual classes on Chest X-ray14 data set: for A/B, A is the value computed for the proposed technique, B is the value of ‘Baseline’ that uses transfer learning with DenseNet201

Class	Spec.	Sens.	F1-Score	Acc.
Atelectasis	0.96/0.94	0.61/0.08	0.56/0.09	93.46/88.73
Cardiomegaly	0.97/0.75	0.86/0.22	0.72/0.08	96.43/72.27
Effusion	0.97/0.86	0.59/0.06	0.66/0.05	93.36/ 76.60
Infiltration	0.93/0.86	0.36/0.09	0.42/0.09	85.13/74.43
Mass	0.97/0.63	0.67/0.19	0.55/0.03	96.56/62.40
Nodule	0.97/0.96	0.41/0.06	0.33/0.04	96.63/94.83
Pneumothorax	0.94/0.97	0.7/0.02	0.66/0.04	92.10/86.90
Consolidation	0.88/0.99	0.73/0.00	0.47/–	87.30/92.2
Pleural thickening	0.93/0.97	0.46/0.01	0.25/0.01	92.46/94.80
No finding	0.95/0.99	0.52/0.00	0.64/–	80.63/65.13

Tools and resources : For our experiments, we fine-tune our models with NVIDIA GTX 1070 GPU with 8GB RAM. For the pretrained models, we use the ImageNet models provided by MathWorks and fine-tuned them using MATLAB. We use the SPAMS library [35] to implement the OMP algorithm.

### Results on chest X-ray14 data set

We summarize the results of our experiments on Chest X-ray14 data set in Table 2. The table includes the results of ‘Baseline’, which is a DenseNet201 model that is pretrained on ImageNet (source domain) and then fine-tuned on our training set (target domain) by the commonly used conventional Transfer Learning (TL) technique. The ‘Den’, ‘VGG’, ‘Res’ and ‘IV3’ respectively denote the DenseNet201, VGG-16, ResNet50 and Inception-V3 models augmented by our technique for transfer learning. The ‘Proposed’ is the proposed full ensemble technique that additionally uses the dictionaries, as discussed in Sect. 3. The table reports the mean values of Spec., Sens., F1-Score, and Acc. across the ten labels used in our experiments.

To show the contribution of each modified model in our technique, each following row of Table 2 adds a new model to the ensemble, denoted by ‘+’ symbol. We also report the Error Reduction Rate (ERR) and Gain resulting from each constituent of the technique. For a given row, ‘ERR’ is the percentage reduction in the error for the model in that row, as compared to the previous row. Similarly, the ‘$\dagger$Gain’ is the percentage improvement in the ‘$\dagger$Acc.’ for a given row as compared to the last row. These results clearly show a consistent improvement in the performance of our technique with each additional component. Results of the full technique are given in the last row.

It is worth indicating that in Table 2, the ‘Baseline’ was chosen after testing conventional transfer learning with DenseNet201, VGG-16, ResNet50 and Inception-V3 separately. We chose DenseNet201 as it performed the best. Despite that, the performance was not acceptable for the challenging radiography test set used in our experiments. The baseline model was not able to generate a single TP prediction of two classes (details below), which also resulted in undefined F1-Score of the model. The proposed augmentation of DenseNet201 led to a significant performance gain. Recall that this gain results from multiple factors, including; extra input layers, larger input image size, increasing channels of input with end-to-end trained layers, and adopting hierarchical procedure for model induction. Owing to the diversity of the chosen models, each new augmented model is able to make an explicit contribution in the final performance, which is further boosted by the dictionary ensemble. We also report the computation time for all the networks for our approach in Table 3. Information on the used computational resources is already provided in Sect. 4.3.

In Table 4, we show detailed results on individual classes on Chest X-ray14 data set. The table also includes the results of ‘Baseline’ for reference. It can be noticed that due to large true negative predictions, the baseline is often able to show good specificity as well as the overall accuracy. However, the sensitivity and F1-score of the baseline remain below the acceptable range. In our experiments, the baseline transfer learning is not able to predict even a single true positive for Consolidation and No Finding. This resulted in an un-defined F1-score. Note that ERR and Gain are defined w.r.t. the baseline. Hence, no values of these metrics are reported in the table. Our technique is able to provide acceptable results across nearly all classes. We emphasize, both baseline and our technique use exactly the same training data to achieve the reported results.

**Table 5:** Results summary on COVID-19 data set: dense denotes DenseNet201 augmented with our technique. Similarly, ‘VGG’, ‘Res’ and ‘IV3’ are augmented versions of VGG-16, ResNet50 and Inception-V3 using our method

Model	Spec. $\%$	Sens. $\%$	F1 $\%$	Acc. without Dict.$\%$	Acc. with Dict. $\%$	$\dagger$ Acc. without Dict.$\%$	$\dagger$Acc. with Dict.$\%$
Dense	98.11	96.21	96.20	97.47	97.47	96.21	96.21
VGG	98.86	97.73	97.74	96.97	98.48	95.45	97.73
Res	97.73	95.45	95.48	94.44	96.97	91.67	95.45
IV3	96.59	93.18	93.08	95.45	95.45	93.18	93.18
Ensemble	99.24	98.48	98.49	98.99	$\varvec{99.49}$	98.48	$\varvec{99.24}$

‘Ensemble’ is the ensemble of the four models. ‘Acc.’ denotes the accuracy for binary classification and ’$\dagger$Acc.’ is the accuracy for multiclass classification

**Table 6:** Results of individual classes on COVID-19 data set

Class	Spec.$\%$	Sens.$\%$	F1$\%$	Acc.$\%$
Covid-19	100	100	100	100
Pneumonia	98.86	100	98.88	99.24
Normal	100	97.73	98.88	99.24

### Results on COVID-19 data set

To establish the efficacy of our technique for a contemporary practical problem, we tested our proposed method to classify COVID-19 cases from chest X-ray images. The results of our experiments on COVID-19 data set are summarized in Table 5. The results are reported for DenseNet-201, VGG-16, ResNet50, Inception-V3 and their Ensemble. Note that, for each of the individual networks, we have used our hierarchical transfer learning technique to get the improved performance. The performance is further boosted with the help of the dictionary. To show the explicit contribution of the dictionaries, we include separate columns for the results achieved by including the dictionaries. We report the both binary and multiclass classification accuracies in the table. It can be observed that both accuracies achieve an overall gain with dictionaries.

In Table 6, we show the average individual results for all three classes in the COVID-19 data set, i.e. COVID-19, Pneumonia and Normal. It is worth emphasizing that the number of reliable labelled COVID-19 X-ray images is very limited and we have used 132 test images. In Table 7, we compare the results of the proposed technique with the related studies using deep learning on similar COVID-19 data sets. Besides the techniques, the table also reports the number of samples used for each technique for the individual classes. These samples are used to train the computational models, whose architecture is also mentioned. As can be seen, the proposed technique performs exceptionally well for the limited amount of data that it uses, achieving the accuracy of 99.49$\%$ for the binary classification task. The promising results of our transfer learning strategy with deep pretrained models in detection of COVID-19 from chest X-ray images indicate that the load of physicians can eventually be reduced reliably with such computer-aided diagnostic techniques.

**Table 7:** Comparison of the proposed technique with other deep learning techniques for COVID-19 diagnostic using chest X-ray images

Study	No. of cases	Architecture	Data set	Accuracy$\%$
Ioannis et al. [5]	224 COVID-19, 700 Pneumonia, 504 Healthy	VGG-19	[4, 11, 24]	98.75
Wang et al. [61]	53 COVID(+), 5526 COVID(−), 8066 Healthy	COVID-Net	[11, 32, 54, 59]	92.4
Ozturk et al. [40]	125 COVID-19, 500 Pneumonia, 500 No finding	Dark COVID-Net	[11]	98
Asif et al. [25]	1300 images of COVID-19, normal, pneumonia	CoroNet	[11, 43]	95
Tougaccar et al. [55]	295 COVID-19, 98 Pneumonia, 65 No findings	MobileNetV2	[11, 54]	99.27
Narin et al. [38]	50 COVID-19, 50 No findings	ResNet-50	[11]	98
Hemaden et al. [18]	25 COVID-19, 25 No findings	VGG-19, DenseNet-121	[11]	90
Sethy et al. [48]	25 COVID-19, 25 No findings	ResNet-50	[11]	95.38
Toraman et al. [56]	1050 COVID, 1050 No finding	CapsNet	[11, 62]	97.24
Panwar et al. [41]	192 COVID-19, 145 No findings	nCOVnet	[11]	97.62
Ucar et al. [58]	76 COVID-19, 1583 normal, 4290 pneumonia	Bayes-SqeezeNet	[11, 23]	98.3
**Proposed**	219 COVID-19, 219 Viral Pneumonia, 219 Normal	Augmented	[11, 54]	$\varvec{99.49}$

## Conclusion and future work

We presented a novel method to improve the performance of transfer learning when the target domain data is not only scarce, but it also has a slightly different modality. Our technique uses an ensemble of deep learning models that are modified and hierarchically fine-tuned to the target domain. Our method takes additional help from dictionary learning—a representation learning framework. We tested our technique by using pretrained models of natural images (i.e. ImageNet) and transferring them to the domain of chest radiography images. We showed that whereas limited data of the target domain remain insufficient to achieve acceptable performance under conventional transfer learning, our technique is able to provide significant performance improvement for the problem. Our results provide a conclusive evidence of the possibility of accuracy gain by allowing for additional model complexity. In the future, we intend to improve our framework by including more recent and accurate models of the source domain and constructing dictionaries directly from the training data instead of using deep features as dictionaries.
